# Supplementary material for: The Aerodynamic Effect of Biomimetic Pigeon Feathered Wing on a 1-DoF Flapping Mechanism
Source: Biomimetics (Basel). 2024 Jan 5;9(1):36. doi: 10.3390/biomimetics9010036 (PMC10813536; doi:10.3390/biomimetics9010036)

Supplementary Material

Flapping mechanism

To convert the constant rotational motion of the driving motor into the flapping motion, this flapping mechanism design utilizes a cylindrical cam-driven transmission pin for linear reciprocating motion, synchronously driving the rotation of the two-wing slider-arm assemblies to complete the flapping motion. The schematic diagram of the mechanism is shown in Figure S.1. On the left side is the cylindrical cam, with its rotation axis parallel to the z-axis. In the center, the vertical slider serves as the transmission pin, constrained by the mechanism to allow only vertical translational motion, which in turn drives the right-side slider-arm assembly and the wing assembly. During the flapping process, the horizontal distance 'd' between the slider and the shoulder joint axis remains a fixed value.

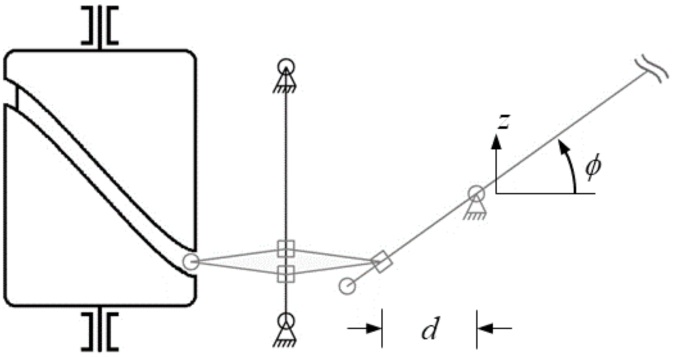

Figure S1. Sketch of the of the flapping mechanism.

The relationship between the flapping angle (ϕ) and the height of the transmission pin (z) is given by Equation S1, where ψ represents the phase angle of the reciprocating flapping motion (Figure S2). By designing the cam angle displacement to be consistent with the flapping phase angle, the cam profile equation can be expressed as Equation S2. In this study, the flapping amplitude (Φ) is designed to be 45°, and the horizontal distance, d, between the shoulder joint axis and the transmission pin is 14 mm.

$$\phi = \arctan\left(-\frac{z}{d}\right) = \Phi \sin(\psi) \tag{S1}$$

$$z(\psi) = -d \tan\left(\Phi \sin(\psi)\right) \tag{S2}$$

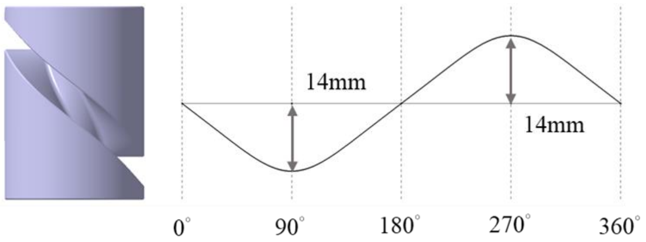

Figure S2. The profile of cylindrical cam.

The overall mechanism is shown in Figure S3, where each wing is driven by a cylindrical cam, and below the cam, a transmission gear is installed. Both wings are synchronized by the same double-stage reduction gear, ensuring symmetrical movement of the left and right wings. Behind the double-stage reduction gear is a cycloidal drive gearbox, which provides a reduction ratio of 15. Combined with the other transmission gear arrangements, the final reduction ratio of the entire mechanism is 14.34. Figure S4 is an exploded view illustrating the assembly of the entire transmission mechanism.

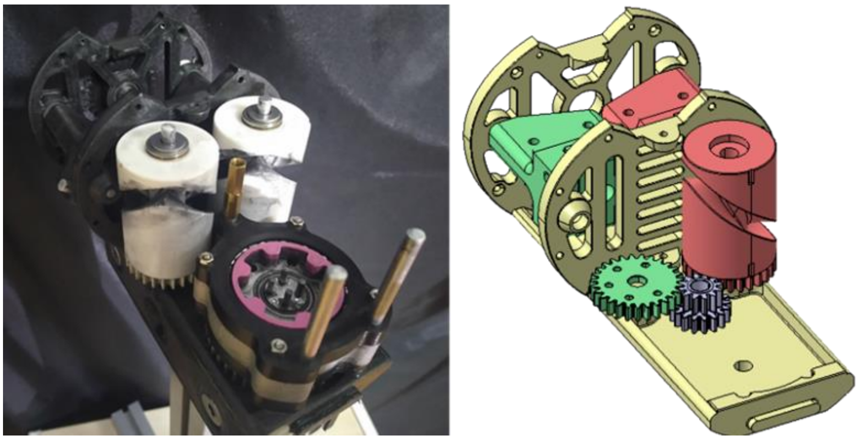

Figure S3. Internal transmission mechanism of the fuselage.

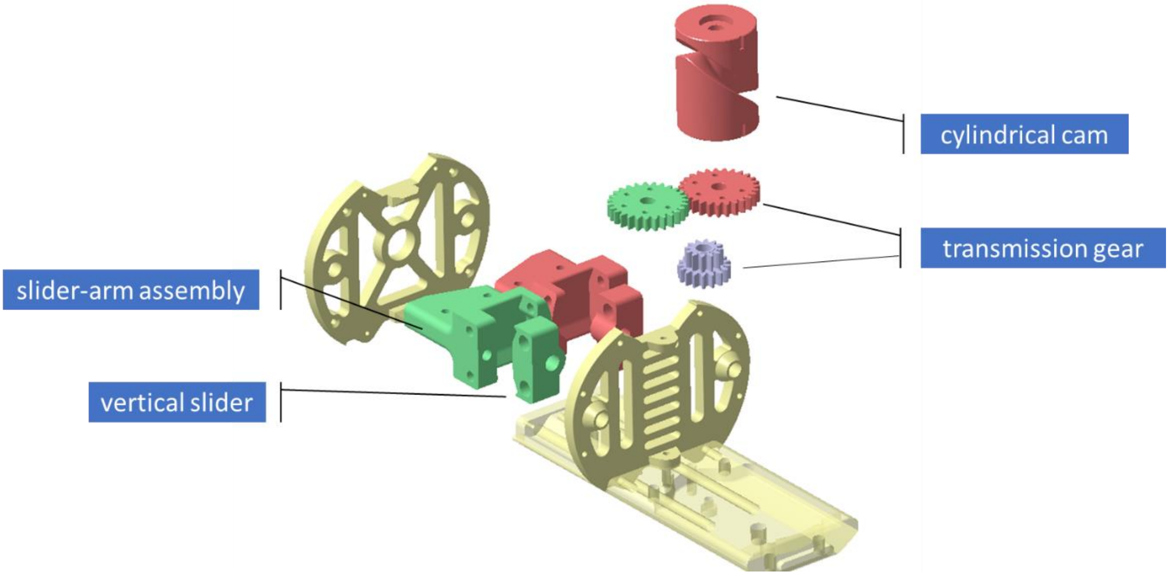

Figure S4. Exploded view of internal transmission mechanism.

Images of different feathers during the flapping process

Frame 8(0) marks the end of the upstroke and the beginning of the downstroke, with each frame sequentially spaced at 1/8 of the flapping cycle.

Real feather:

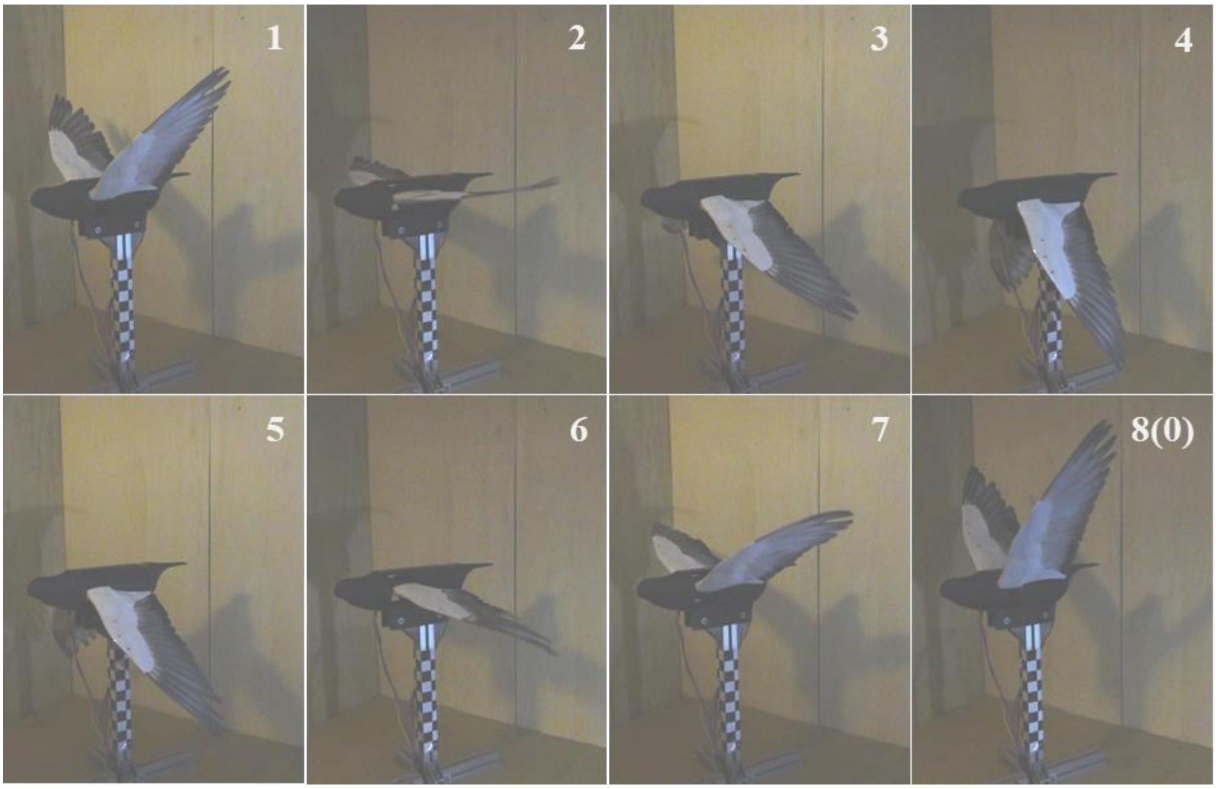

PLA artificial feather:

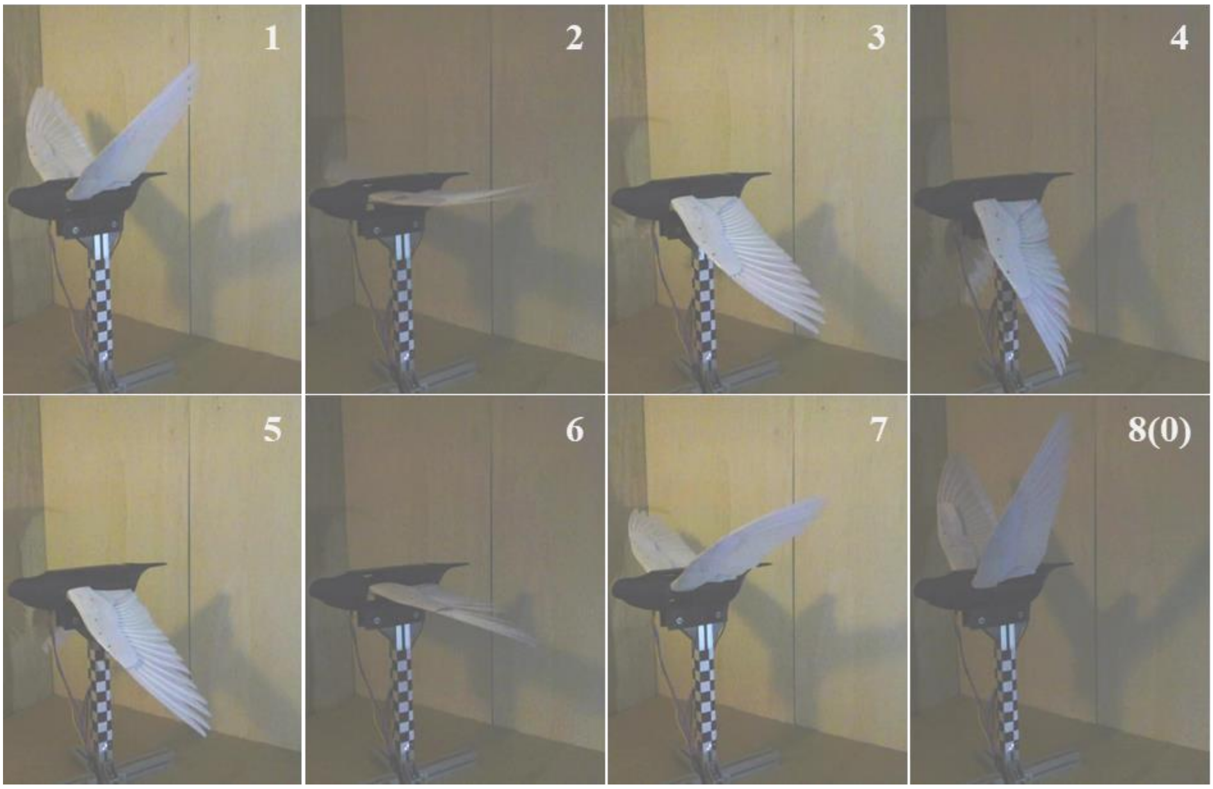

PETG artificial feather:

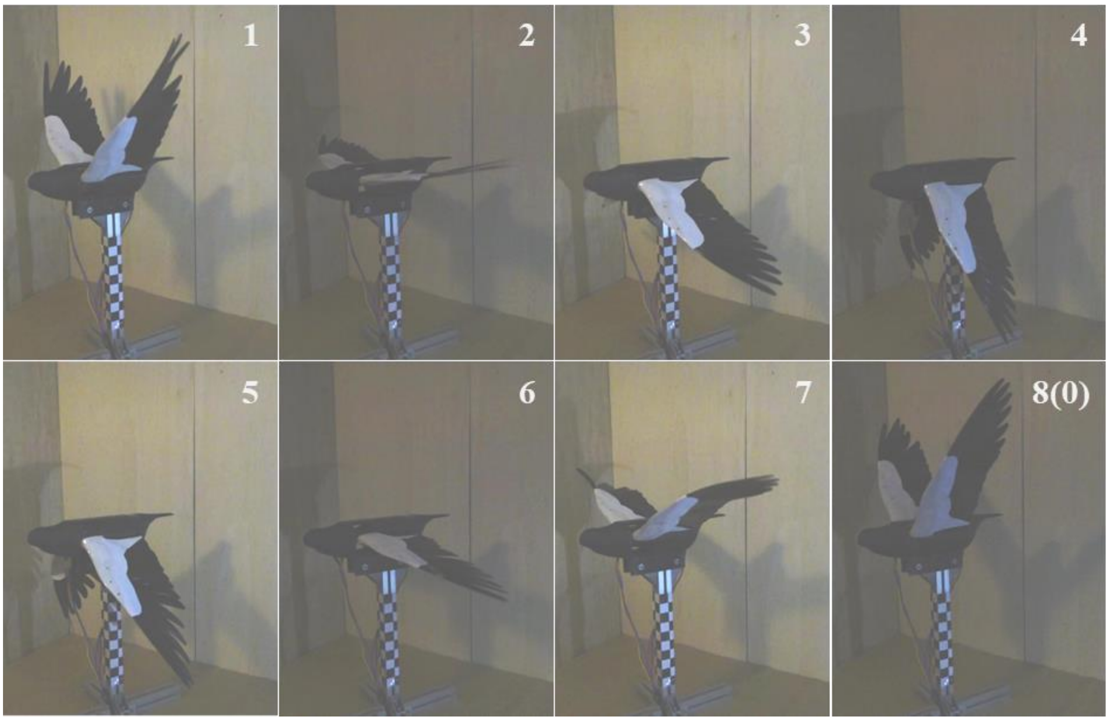

Supplement: Supplementary file 1 [file biomimetics-09-00036-s001.zip › biomimetics-2663968-supplementary.pdf]
